# Editorial: Metaphor studies: theories, methods, approaches, and future perspectives

**DOI:** 10.3389/fpsyg.2024.1520900

**Published:** 2024-11-28

**Authors:** Ilaria Rizzato

**Affiliations:** ^1^Department of Modern Languages and Cultures, University of Genoa, Genoa, Italy; ^2^CIRM: Inter-University Centre for Metaphor Research, University of Genoa, Genoa, Italy

**Keywords:** metaphor, figurative language, metaphorical thought, abstract conceptualization, embodied metaphors, metaphor in discourse, metaphor variation

## 1 Introduction: aims and scope

This Research Topic seeks to provide an open and inclusive space for the discussion of Metaphor Studies as a plural, diverse research area encompassing a wide range of disciplines as well as a number of theories, approaches, and methods. It aims to enrich and expand on the platform provided by Lakoff and Johnson's Conceptual Metaphor Theory (Lakoff and Johnson, 1980) and by the responses and further developments that the CMT has elicited in the field over the past few decades, including the Dynamic Systems Approach (Gibbs, 2008), Deliberate Metaphor Theory (Steen, 2008, 2023), Kövecses' studies on embodiment and context (Kövecses, 2000, 2015), and the theory of conflictual concepts (Prandi, 2012, 2017), to mention but a few of the works that have elaborated on the cognitive theoretical paradigm and proposed further ways of conceptualizing, interpreting, and researching metaphors. Based on these premises, this Research Topic attempts to bring the discussion forward by promoting interdisciplinarity, diversity, and depth. In this connection, this Research Topic showcases a number of different conceptual and methodological approaches to Metaphor Studies, pursued through a variety of theoretical and analytical tools, and applied to a wide array of social, cultural and linguistic domains, with the aim of favoring cross-fertilization among the many research areas involved, thus offering a comprehensive account of existing research scenarios and their possible future developments.

The next section provides a brief overview of the contributions to the Research Topic. In order to highlight the main points of contact among the articles, five major thematic areas have been identified, including the conceptualizing potential of metaphors, metaphor comprehension in learning and teaching processes, semantics and metaphor processing, metaphor and multimodality, and new contributions to metaphor theory. The articles have been assigned to one thematic category for the sake of simplicity and tidiness, but this classification is not meant to be exhaustive of the possible similarities, analogies, and interconnections that may emerge from a full reading of each contribution.

## 2 Contributions to the Research Topic *Metaphor studies: theories, methods, approaches, and future perspectives*

A total of 22 articles contributed to this Research Topic, bringing together different strands in a common search for methods and approaches that might pave the way for positive developments in the research field of Metaphor Studies. A solid starting point in this direction is Zhao et al.'s bibliometric analysis of conceptual metaphor research, which shows the steady increase in such studies over the past 20 years and points to the many ramifications and re-elaborations of Conceptual Metaphor Theory.

The potential of metaphor for conceptualizing abstract, intangible and/or complex entities is well-represented in this Research Topic, which covers topical fields of inquiry such as health and social discourse in addition to areas of vast consequence in linguistics and psychology such as the notion of time and its relationship with space. Shi and Khoo, for example, analyzed how depression sufferers conceptualize their experience metaphorically in Chinese in a self-constructed corpus of texts produced by online health communities. Xu examined COVID-19 metaphors used on Twitter and Weibo and compared the different outcomes emerging in English and Chinese utterances. Negrea-Busuioc looked at the criticism raised by the use of a specific COVID-19-related metaphor on Facebook due to the different connotations and projections it may activate. Liu and Chen investigated the role of metaphors in shaping people's attitudes toward the risk of telecom fraud through an experiment confronting them with different framings. Zhang and Yang conducted a corpus-based analysis of economic texts to find out that the landslide concept prevails in terms of impact over other disaster notions in communicating economic crises. Feist and Duffy explored two widely studied metaphors for time, moving time and moving ego, to point out the limitations of CMT unifying theory and to put an emphasis on variation, individuality and context in metaphor use. Park et al.'s experiment, in which Arabic-English bilingual participants were required to arrange cards so as to tell a story in chronological order proves that the writing direction of the language being used affects the representation of time in terms of space.

Metaphor as a conceptualizing tool also features prominently in the study of comprehension and learning and teaching processes. In this connection, Giuliani looked at the metaphors used by teachers and educators to elicit positive, resilient responses to the pandemic in primary and secondary schools in Italy. Cheng et al. investigated the metaphorical comprehension of Chinese children aged 5–8 years by analyzing response times and accuracy rates in processing metaphorical and literal sentences and found that metaphorical ability improves with age. Yu et al. used event-related potentials to compare brain responses in the comprehension of both novel and conventional metaphors and novel and conventional similes and interestingly found no significant difference between novel and conventional similes. Tay subverted the static target is source formula and invoked a dynamic approach to metaphor use, for which he devised a pilot study among statistics students consisting of transforming target domains into source domains in unconventional, creative metaphors. Gebbia considered the translation strategies spontaneously adopted by 73 translation students to render creative metaphors and their textual patterning and the related output. The findings suggest that isolated, non-conflictual metaphors do not pose any problems, whereas the diverse patterns of conflictual ones result in problematic outcomes, lacking connotative elements, figurative diversity, and cohesion.

Questions of meaning-making through figurative language are also prominent in the articles dealing with semantics and metaphor processing. Al-Azary and Katz studied the role of two semantic richness variables in metaphor production: semantic neighborhood density (SND), which measures the proximity of a word and its associations in semantic space, and body-object interaction (BOI), which reflects the ease with which a human body can motorically interact with a word's referent. They found that participants in their experiment appeared to try to reduce the overall semantic richness of the metaphors they created. De Backer et al. discussed the possible issues deriving from a quantitative analysis of the semantic field of metaphor-related words through MIP/VU and proposed three methods to extend its protocols to identify metaphorical strings in a more consistent manner. Zhang et al. applied Event Related Potential (ERP) technology to look at Chinese action verbs in their metaphorical sense and literal and abstract verbs. The comparison between the evidence from different sets of verbs suggests that the comprehension of the metaphorical action verbs is based on the semantics of concrete action, which cannot be said for literal and abstract verbs.

The abstract and concrete spheres simultaneously come into play when the object of analysis is a multimodal metaphor. Zhong et al. conducted a bibliometric analysis of the field of multimodal metaphor over the years 1977–2022, with a focus on 397 relevant publications retrieved from the Web of Science Core Collection through the visualization tool VOSviewer and identified topical moments and themes in this area. Guerrieri et al. sought to provide a definition of artistic metaphor and outlined the multimodal properties of metaphor in artistic environments. They tested their definition through a corpus of artworks by prominent Sardinian artists to show that the visual, tactile, and auditory components of the pictures can boost effective comprehension of figurative meaning. Borgogni reassessed Renaissance emblematics through the lens of recent metaphor theory to cast light on the complex and refined multimodal patterns offered by the interplay of visual and verbal text with figuration. His analysis subverts the traditional view of emblems as marginal by-products in Renaissance texts, revealing that metaphorical concepts in emblems predominantly rely on conflict rather than similarity.

More traditional views are questioned in the final section, where major theoretical tenets on the nature and behavior of metaphors are addressed and explored. Garello and Carapezza challenged what they call the “Natural Kind Assumption,” that is, the widespread notion that despite their differences, metaphors share many properties and that a theory of metaphor should capture such essential properties. In their article they subverted this assumption and discussed the main consequences of this view shift on the philosophical plane. Colston discussed the conceptualization of metaphor as an entity relying on the dual structure of source and target and traced the success of such a conceptualization back to the human need for shared interpretative patterns for complex meanings. Thus, simpler dual frameworks tend to prevail, despite the many advantages that multiple structures can provide in the analysis of complex phenomena. Finally, Steen's article looked at the most recent developments in Deliberate Metaphor Theory, based on the identification of metaphors that are subject to both a non-deliberate and a deliberate reading, so that they can produce different outcomes in communication, also depending on participant-related circumstances. Participants' responses are also relevant to the other finding this article exposes: different types of metaphors elicit different types of thinking, particularly fast and slow thinking, the former being associated with less processing effort and with non-deliberate, conventional metaphors, the latter with more processing effort and deliberate, unconventional metaphors, or very complex instances of metaphors that require considerable interpretive commitment to be processed.

Among the declared goals of the latter article is the search for principles that can serve as a unifying function in metaphor theory and research. Similarly, this Research Topic attempts to bring together different theories, methods and approaches in one place for discussion and dialogue, which may act as a starting point for future developments.
